# Development of a Biosensor for Detection of Pleural Mesothelioma Cancer Biomarker Using Surface Imprinting

**DOI:** 10.1371/journal.pone.0057681

**Published:** 2013-03-13

**Authors:** Aabhas Mathur, Steven Blais, Chandra M. V. Goparaju, Thomas Neubert, Harvey Pass, Kalle Levon

**Affiliations:** 1 Department of Chemical and Biological Sciences, Polytechnic Institute of NYU, Brooklyn, New York, United States of America; 2 Kimmel Center of Biology and Medicine at Skirball Institute and Department of Pharmacology, NYU School of Medicine, New York City, New York, United States of America; 3 Department of Cardiothoracic Surgery, NYU Medical Center, New York City, New York, United States of America; Johns Hopkins University, United States of America

## Abstract

Hyaluronan-linked protein 1 (HAPLN1) which has been shown to be highly expressed in malignant pleural mesotheliomas (MPM), was detected in serum using an electrochemical surface-imprinting method. First, the detection method was optimized using Bovine serum albumin (BSA) as a model protein to mimic the optimal conditions required to imprint the similar molecular weight protein HAPLN1. BSA was imprinted on the gold electrode with hydroxyl terminated alkane thiols, which formed a self-assembled monolayer (SAM) around BSA. The analyte (BSA) was then washed away and its imprint (empty cavity with shape-memory) was used for detection of BSA in a solution, using electrochemical open-circuit potential method, namely potentiometry. Factors considered to optimize the conditions include incubation time, protein concentration, limit of detection and size of electrode. Matrix assisted laser desorption ionization-time of flight mass spectrometry (MALDI-TOF MS) was used to confirm selectivity of imprints. With the obtained imprinting control parameters, HAPLN1 was imprinted in duplicate and the detection of spiked HAPLN1 was successfully conducted in serum.

## Introduction

A possible cure of cancer would be more likely if the disease could be detected early for optimizing the therapeutic procedures. Extra-cellular matrix (ECM) has its importance not only for the physical support of various cells but also in cell-cell interactions, cell signaling and cell repair processes. Therefore ECM proteins themselves present a potential monitoring target reflecting the ongoing changes. If the changes occur due to the presence of a disease such as cancer, the detection of these changes would be a presumptive disclosure of cancer before any symptoms appear. As an example, heparanase enzyme [1], an endo-ß-glucoronidase, is a multifunctional enzyme influencing key steps of development of numerous types of cancers such as gastric [2], non-small lung [3], esophageal [4], head and neck [5], breast [6] and pancreatic [7] cancers as examples.

Heparanase cleaves enzymatically heparin sulfate and with serine proteinases and metalloproteinases plays a crucial role in the extracellular matrix remodeling associated both with in situ tumors growth and the metastatis-related invasion of cancer cells. The enzymatically inactive form of heparanase has been shown to affect cell signaling, and thus increasing cancer growth with upregulation of several genes expression [8]. Commercial biomarkers for monitoring asbestos-induced mesothelioma activity are mesothelin and soluble mesothelin related peptide (SMRP). Osteopontin (OPN) and CA125 have similarly been related to cancer-linked heparanase activity in addition to hyaluronan, TRA and ferritin. The ECM remodelling events may certainly be a target for diagnosis of the early stages of cancer invasion [9] as well as targets for anti-cancerous treatments. Hyaluronic acid (hyaluronan, hyaluronate, HA) is a linear, very high molecular weight glycosaminoglycan, is one of the most abundant ECM proteins. HA has been shown to exist at high levels in colorectal, epithelial ovarian, and breast cancers [10]–[12] and also tumor growth inhibition has been evidenced when HA binding motifs have been affected [13]. HA overexpression is greatly dependent on the CD44, so the regulation of bladder cancer growth, invasion and angiogenesis was proven to occur through the CD44 by HA synthase-1 expression [14]. As the HA expression seems to be clearly elevated at the appearance of cancer it is not surprising that Ivanova et al have shown that also HAPLN1 protein, which helps to link proteoglycans to a HA core, has been shown to be elevated in cancer cells.

Malignant pleural mesothelioma is a rare malignancy caused by asbestos exposure, which has a poor prognosis. Cartilage link protein HAPLN1 is being studied as a candidate biomarker, found to be overexpressed in mesothelioma stage 1 along with a highly expressed cancer biomarker osteopontin (OPN1) located in the same gene set [15]. Immunoassays are commonly used for detection of cancer biomarkers as they show superior selectivity, but can be time consuming and expensive. The biological features of markers such as heparanase emphasize the importance to design cost effective methods allowing the early detection of such biomarkers in serum and tissues both for diagnostic and prognostic purposes as well as for monitoring cancer treatments.

Also detection of very low concentration of such biomarkers with ELISA tests is difficult. Therefore, there is an urgent need for development of new technologies, which are rapid and very sensitive to detect low concentrations of biomarkers. Early diagnosis will provide more time for treatment of cancer, which is crucial to save a life. Molecular imprinted polymers are being considered as a suitable replacement for antibody-based systems as biosensors, due to better stability and reusability of the bio-recognition surfaces [16]. Biosensors are composed of two components, bio-receptor element and signal transduction component. A bio-receptor surface made with molecular imprinted polymers would be two orders of magnitude cheaper then antibodies. As price of the hydroxylated alkane thiol polymer would cost about $0.2–0.6 mg^−1^ compared to antibodies, which vary depending on the target. Antibodies generated for targeting human HAPLN1 protein would cost about $900–$1000 mg^−1^. Even low density of such antibodies on immunoaffinity cartridges have higher price than molecular imprinted polymers, costing between $10 to $100 mg−1. High price of HAPLN1 antibodies increase the cost of HAPLN1 Elisa kits commercially available to $10/test. Whereas an imprinted gold coated sensor chip (1 cm×1 cm) would cost approximately $ 1.2/test and can be used repeatedly for multiple assays unlike antibodies, which cannot be reused.

In our study we aim to develop a tool for early detection of biomarkers. Target protein molecules co-adsorb on the surface of gold electrode with the thiols and they together form a well-packed monolayer. Removal of the protein molecules from the SAM form imprint cavities and the functional hydroxyl head group of the thiols provide specificity to cavity. The complementarity in size, shape and hydrophobicity provides affinity towards the same molecule, which has been imprinted.

The imprinted electrode is then used to assay the template in buffer using potentiometry. As the charged protein molecule in buffer binds to the cavities it changes the potential of the sensing electrode. Synthesis of the imprint surface is straightforward and does not require any expensive technology for preparation. It is an old but promising technology, which has been developing with the challenges it has faced in the past.

**Figure 1:**
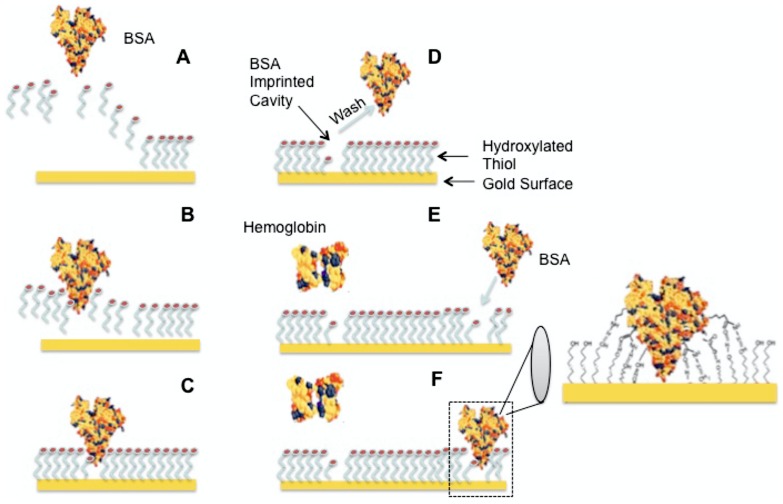
2D Imprinting process and detection of analyte protein. (A–C) Insertion of the protein within the SAM. (D) Wash. (E–F) Binding of the BSA analyte protein, hemoglobin as a non-specific control.

In 1930 a Soviet scientist M.V Polyakov demonstrated that silica gel prepared in the presence of a solvent additive showed preferred binding to the same solvent [17]. Linus Pauling and Frank Dickey published in 1949 that silica shows specificity for dyes imprinted on the surface [18]. Major breakthrough in molecular imprinting took place in 1972 when Gunter Wulff prepared molecular imprinted organic polymers using the covalent binding approach, which can differentiate between enantiomers of glyceric acid [19]. Wulff et al. used an approach, which became the most famous for molecular imprinting studies during the 70's until another theory was introduced in 1981 by Mosbach and coworkers. They created molecular imprints based on non-covalent binding with the template [20]. It was the simplicity of this approach that triggered an explosion in 90's with molecular imprinting polymers. We have used non-covalent binding approach introduced by Wulff et al. to imprint the HAPLN1 biomarker by using hydroxyl functional groups on thiols which form weak interactions with the protein and a well fitted mold around the same. This provides more specificity to the cavity, thus making the removal of HAPLN1 from the cavity easier. The imprint conditions were optimized for potentiometry using BSA (66 kDa) protein (Figure 1), due to its similarity in size with HAPLN1 cancer biomarker (65 kDa). We tested the specificity of the imprinted cavities, using MALDI-TOF, by performing tests to show that a relatively smaller protein Myoglobin (17 kDa) does not bind to BSA imprints.

## Materials and Methods

### Principle of potentiometric detection

In our technology, we have used potentiometry for detection of target analyte HAPLN1. A potentiometer measures the potential difference between a reference electrode (Ag/AgCl) and the working electrode. Analyte binds to the bioreceptor surface on the working electrode in a buffer solution, resulting in a change in potential difference between the two electrodes. This change is then measured by the system. Smaller biomacromolecular ions do not result in a voltage as they remain in equilibrium with the electrolyte.

The strong voltage response results from the macroions binding via extensive macromolecular hydrogen bonding around the imprint On the working electrode (Gold coated silicon chip), a large biomacromolecule recombinant HAPLN1, 65 kDa size protein assembles with thiols, which form an organized, dense SAM around the analyte. Thiols have the tendency to self assemble on the gold surface as the sulfur group on the tail end of thiols forms a sulfur-metal bond with gold [21]. Organized SAM is an important ‘sealant’ against interferrents and pinholes for parasitic current. The macromolecule is then washed from the surface, which results in formation of cavities. The imprinted electrode acts as the bioreceptor element of the biosensor. Thus imprinted electrode works with same principles as commercial ion selective electrodes, which have a specific ionophore to recognize the analyte ion. We show that potentiometry can be used with good sensitivity and selectivity to detect the target cancer biomarker in buffer solution and in spiked serum. The electrochemical response obtained with this technique is due to the potential change after the binding of the template protein. The change in potential is measured with respect to a reference Ag/AgCl electrode [22], [23].

### Materials required for electrochemical measurement

Bovine Serum Albumin (BSA) from bovine blood (Mw = 66.3 K), Myoglobin from equine skeletal muscle (Mw = 17.6 K), 11-mercapto-1-undecanol (97%) (thiol), absolute ethanol, methanol, and isopropanol were purchased from Sigma Aldrich (St. Louis, MO, USA). Human HAPLN1 (64.68 kDa) full-length ORF (AAH57808, 1–354 a.a.) recombinant protein with GST-tag at N-terminal was purchased from Abnova Corporation (Walnut, CA) and stored at −80°C. All the proteins and chemicals were used as received. Two kinds of electrodes were used as the sensing electrode: the gold (50 nm) sputter coated silicon wafer (1 cm×2 cm) and gold electrodes (2 mm^2^) purchased from BASi (West Lafayette, IN). The potentiometric measurements were made in 10 ml 1X PBS in 20 ml glass vial, equipped with a magnetic stirrer. When diluted to a 1X concentration, 10 X phosphate buffered saline solution purchased from Sigma Aldrich (St Louis, MO) yield a phosphate buffer concentration of 0.01 M and a sodium chloride concentration of 0.154 M with pH 7.4. The two-electrode system consisted of an Ag/AgCl as the reference electrode and silicon coated gold chip gold electrode as the working electrode. The potentials of the working electrode against the reference electrode were measured with an Accumet AR15 potentiometer purchased from Fisher Scientific (Pittsburgh, PA) and EMF 16 channel Electrochemical Interface purchased from Lawson Labs (Malverin, PA)

### Materials required for MALDI TOF

Gold target plate was purchased from Bruker Daltonics (Billerica, MA). TA solution (0.1% trifluoracetic acid/acetonitrile, 2∶1,vol∶vol) and Sinapinic acid were purchased from Sigma-Aldrich (St. Louis, MO) for the matrix preparation to immobilize the protein bound on the surface of the target plate. Bruker Autoflex MALDI-TOF purchased from Bruker Daltonics (Billerica, MA) was used to determine the specificity of protein binding to respective imprints on the surface of the target plate. Chemical resistant conformable PTFE (Teflon) Tape was purchased from CS Hyde company (Lake Villa, IL). Nitrogen gas was supplied by Presto-O-Sales and Services (LIC, NY).

### BSA Sensor Fabrication

The silicon chips were cleaned by sonication in de-ionized water, methanol and isopropanol. Silicon chips were allowed to dry overnight and then sputter coated with gold. The gold coated chips were then washed with de-ionized water and dried with pure nitrogen gas. Similarly gold electrodes were cleaned with 0.1 and 0.05 micron alumina and then sonicated with de-ionized water to remove any bound alumina particles. Since proteins denature in non-aqueous solution and thiols show poor solubility in aqueous solution proteins were dissolved in de-ionized water whereas thiols were dissolved in absolute ethanol. Compromising mixed solutions of 19∶1 and 18∶2 (water∶ ethanol) with final thiol concentration of 0.1 mM and 0.2 mM were prepared in clean glass containers and comparatively studied to achieve good solubility of thiols and avoid protein precipitation. The gold coated electrode is then dipped inside the mixed solution to form the self assembled monolayer of protein and thiol. The head space of the container is filled with inert nitrogen gas and covered with parafilm. Incubation time of the chip in the mixed solution was varied for a better imprinted surface. The gold chip is then thoroughly rinsed with de-ionized water to remove the bound protein. Also different protein concentrations of 30 µg/ml, 10 ng/ml and 1 ng/ml were used to form the imprints and the binding results were compared. The electrodes prepared were then used to check binding response with variable BSA concentrations in buffer solution.

### Preparation of Imprints on MALDI-TOF target plate

**Figure 2:**
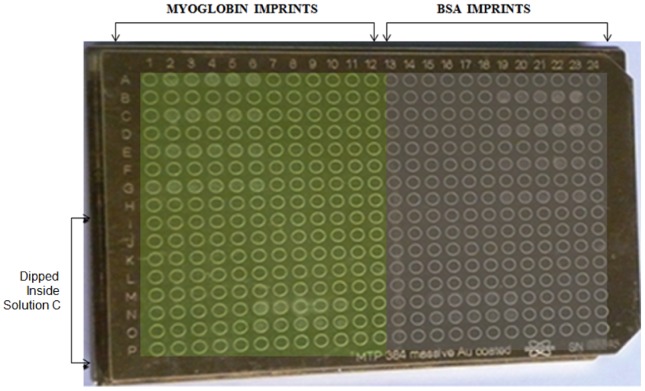
Maldi target plate imprint design. Columns (1–12) for myoglobin and columns (13–24) for BSA imprinting sites. Rows I to P were immersed in the analyte solution C containing both analyte proteins.

Except the surface containing 384 wells, the rest of the gold target plate (8 cmx12.5 cm) was covered with nonreactive Teflon adhesive tape. The gold target surface was cleaned with ethanol and de-ionized water. Controlled flow of inert nitrogen gas was used to dry the target surface. Solution A was prepared with a 19∶1 mixture of 16.17 mg Myoglobin (i.e 1.7 µM) in 540 ml de-ionized water and 0.22 g 11-mercapto-1-undecanol (i.e 2 mM) in 540 ml absolute ethanol. This solution was formed to create myoglobin imprints on one half of gold target plate. Solution B contained 19∶1 ratio mixture (water∶ ethanol) of 16.17 g bovine serum albumin (i.e 0.432 µM) dissolved in 540 ml de-ionized water and 0.22 g 11 mercapto1-undecanol (i.e 2 mM) in 540 ml absolute ethanol. This solution was prepared to create BSA imprints on the other half of gold target plate. Half of the Au plate was immersed in Solution A for 12 hrs to co-absorb the protein and thiol, whereafter it was dried with pure nitrogen gas. Similarly the other half of the gold target plate is immersed in solution B for 12 hrs and dried with pure nitrogen gas. Protein and thiol concentrations were kept same as used for imprinting on gold electrode for potentiometry. Cavities that are complementary with the template proteins were created in the monolayer by removing the protein molecules through repetitive rinsing with de-ionized water. Solution C was prepared with equimolar mixture of 1 ng/ml BSA and myoglobin in 540 ml deionized water. This solution was formed to assay protein binding to two different types of cavities on gold target plate surface. Wells on the target surface were divided into four quadrants (Figure 2) and wells in row I(1–24) to P(1–24) were dipped for 10–15 minutes in solution C. The surface was rinsed gently and allowed to dry using nitrogen gas. Then 1 µl of SA matrix is added to the target and allowed to dry. Well in row A(1–24) to H(1–24) were used for control studies. The target plate was then used for MALDI- TOF analysis.

### HAPLN1 Sensor Fabrication

HAPLN1 protein is over expressed in the majority of mesotheliomas and hence being considered as a potential biomarker for cancer detection. In a clean glass container 20 µg of HAPLN1 (64.68 kDa) protein was dissolved in 10 ml of de-ionized water (i.e 0.03 µM) and 4 mg 11-mercapto-1-undecanol was dissolved in 10 ml ethanol (i.e 0.1 mM). A mixed solution is formed, by dissolving both the solutions in 19∶1 ratio (water∶ ethanol), such that the final concentration of thiol in the solution becomes 0.1 mM. Three gold electrodes A, B and C were dipped in the solution for 12 hours and then the electrodes were rinsed thoroughly with de-ionized water to remove bound protein on the surface.

### Sensor response to HAPLN1 spiked serum samples

HAPLN1-imprinted electrodes were also used for potentiometric response to the cancer biomarker spiked in serum. The 100 nM stock solution in 1∶8 serum and buffer ratio was prepared by diluting 100 µl of human serum with 650 µl of 1X PBS solution and with 250 µl of the commercial 102 µg/mL HAPLN1 solution (i.e. a total of 25.5 µg of HAPLN1). The HAPLN1 concentration in the stock solution was then 6.5 µg/ml (100 nM). 10 µl of solution was used for titration in 10 ml PBS buffer, and the highest concentration of protein is 10^−9^ M. Remaining standards were prepared by maintaining the same dilution rate (1∶8) of serum in buffer but analyte concentration was reduced ten times with serial a 6.5 µg/ml (100 nM) standard spiked solution.

## Results

### Optimization of parameters for BSA Sensor

**Figure 3:**
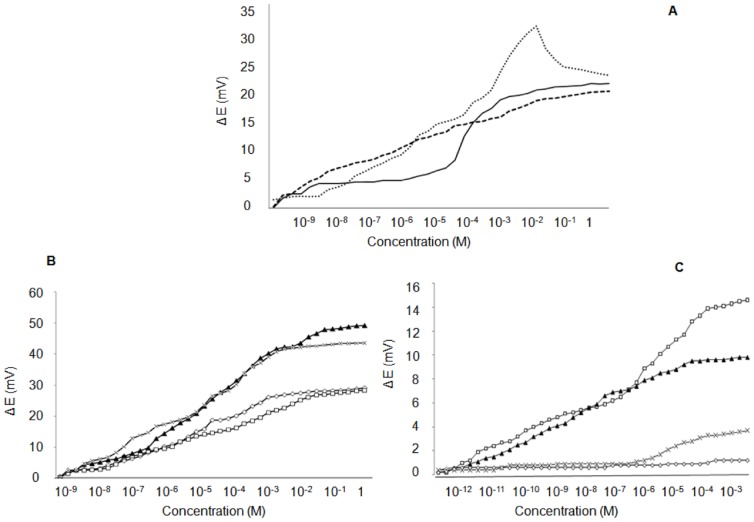
Optimization parameters for BSA imprint. A. Effect of immersion time of 2 hrs (___), 6 hrs (…) and 12 hrs (---). B. Effect of protein concentration of 30 µg/mL (▴, x) and 2 ng/mL (◊,△). C. Effect of protein concentration of 10 ng/mL (□) and 1 ng/mL (▴) and controls on SAM only (x, ◊).

The BSA protein molecules were imprinted with the hydroxyl terminated alkane thiols on the gold surface. As thiols form an organized monolayer with time on the gold surface it was crucial to study the incubation time of the gold coated silicon chip inside the protein-thiol solution to achieve stable imprints. In this experiment the BSA concentration in the mixed solution was 30 µg/ml and Figure 3a shows the effect of incubation time on the stability of the imprints. Three electrodes were prepared by keeping the chips inside the solution for 2, 6 and 12 hours. The mixed solution in this experiment had a ratio of 18∶2 (water∶ ethanol) with 0.2 mM 11 mercapto1-undecanol. All measurements were done in Phosphate buffer saline (pH 7.4) in a 10 ml solution. A logarithmic increase in potential was observed with increasing BSA concentration in buffer only with the electrode, which was incubated for up to 12 hours. The results indicated that more stable imprints were formed when chip were kept in solution for 12 hours. Experiments were then conducted to optimize the conditions to achieve stable protein imprints. The potentiometric response was studied by varying the concentration of BSA imprinted on the electrode to form the cavities. In this experiment two electrodes were prepared using two different concentrations of 30 µg/ml and 1 ng/ml BSA in mixed solutions of protein and thiol.

These mixed solutions were prepared with a 19∶1 ratio (water∶ ethanol) having a final concentration of 0.1 mM 11-mercapto-1-undecanol. The incubation time was kept at 12 hours, as this was shown to support the formation of the most stable imprints.

As shown in Figure 3b, a logarithmic increase of the change in potential takes place with increase in protein concentration. The response of electrode prepared with 30 µg/ml protein imprinting concentration is much higher compared with the other electrode prepared with a lower protein imprinting concentration. This demonstrates that the number of cavities formed on the surface increases as more protein is adsorbed on the surface. The isoelectric point of a protein is defined as the pH at which the net charge on a protein surface is zero. Since BSA molecules have an isoelectric point of 4.7 they have a net negative charge in a buffer solution with pH 7.4. As more charged molecules bind to the electrode surface it leads to a drastic change in potential. The results were successfully repeated for confirmation. Figure 3b also demonstrate a higher potential change with a 19∶1 (water∶ ethanol) ratio when compared to a 18∶2 ratio from Figure 3a. This result indicates that more cavities are formed as the thiol concentration in the solution is reduced. To downscale the process and prepare more sensitive electrodes, low imprinting concentrations of 1 ng/ml and 10 ng/ml were selected based on previous experimental data. Experiments were conducted with gold electrode of much smaller surface area (2 mm^2^). The working electrode was prepared in a 19∶1 (water∶ ethanol) ratio with a final concentration of 0.1 mM 11-mercapto-1-undecanol. As shown in Figure 3c the electrode shows a good binding response of 15–20 mV with very low concentrations of BSA when compared to a low binding response of 2–4 mV observed with prepared control electrodes which only have the SAM on their surface.

### Validation using MALDI-TOF

**Figure 4:**
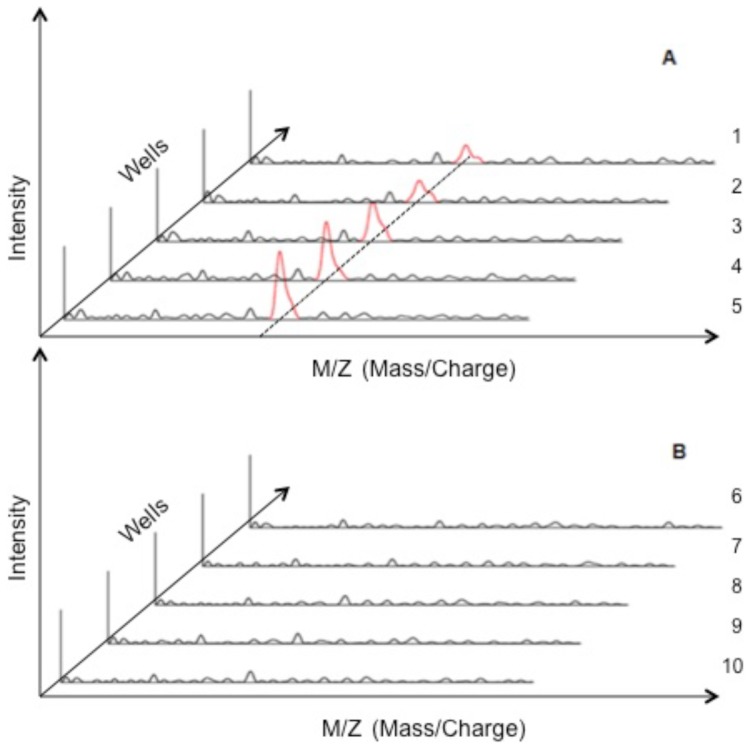
Maldi results for Myoglobin binding to five randomly selected wells. A. Myoglobin imprinted wells and B. BSA imprinted wells.

In this experiment 1 µl of matrix was placed on the top of each well of the imprinted Maldi plate and allowed to dry. Maldi plate analysis software with an algorithm to collect data with a scan rate of 2000 laser shot/s for each spot in a well was selected. Since half of 384 wells on the maldi plate were imprinted BSA and the other half with myoglobin protein, randomly five wells were selected from myoglobin imprinted wells and similarly five were selected from the BSA imprinted wells, to assay the protein bound to the cavities. As shown in Figure 4, three-character symbol m/z in x-axis is used in mass spectroscopy to denote the dimensionless quantity, formed by dividing the mass of an ion in unified atomic mass units by its charge number.

Y-axis show the signal intensity, but instead of using counts per second (cps), the common procedure is to relate the intensity to relative abundance with a use if an internal standard. As shown in Figure 4a myoglobin which has a molecular weight of 17 kDa was observed to be bound to only myoglobin imprinted wells as a peak appears at 17 kDa for each well and no myoglobin was observed in BSA cavities as shown in Figure 4b. BSA was observed to be of too large molecular weight under these ionizations conditions.

### HAPLN1 Sensor

**Figure 5:**
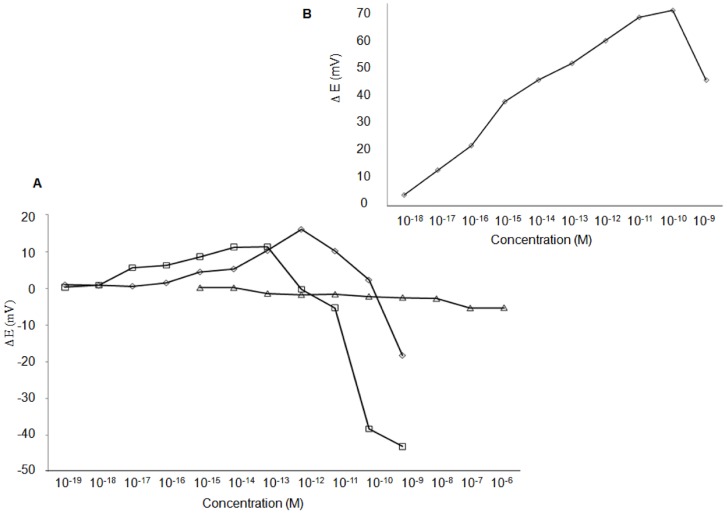
Potentiometric detection of binding response to imprinted electrode. A. HAPLN1 binding to HAPLN1 imprints (two independent experiments), Control BSA response to HAPNL1 imprints. B. Spiked HAPLN1 analyte serum solution to HAPLN1 imprints.

After the BSA studies for optimizing the conditions required for imprinting biomacromolecules, HAPLN1 imprinted electrodes were fabricated for low concentration detection of the biomarker. Three imprinted electrodes were studied to verify the response after detection. Two independent experiments show a drastic 25 mV change in the potential at very low HAPLN1 concentrations of 10^−12^ M as the HAPLN1 was titrated into the buffer solution (Figure 5a). As a control, BSA was also titrated under the same conditions showing a very low potential response at high BSA concentrations. The experiment was then conducted in serum with a spiked HAPLN1 concentration. Figure 5b shows that the imprinted electrode has a high drift in serum but shows a remarkable potential change at 10^−9^ M concentration of HAPLN1 in serum.

## Discussion

The detection of cancer biomarkers with a low cost and easy-to-use method is important for continuous monitoring of multiple markers. HAPLN1 is an example of increasing importance in the field as for instance Nelson et al [24] recently completed a transcriptome analysis on blood vascular (BEC) and lymphatic endothelial cells (LEC) and the clustering analysis showed clearly differential gene expression for BECs and LECs. HAPLN1 was second highest expressed gene in LECs and as tumors take the advantage of these metastatic potential enhancing molecules, the observed high HAPLN1 expression can lead to new therapeutic treatments for metastasis of cancer to lymph nodes. Two-dimensional molecular imprinting technology has been successfully used to imprint and detect small molecules [25]–[29] like peptides but imprinting macromolecules remained a challenging task. Biological macromolecules like proteins have high molecular weight and therefore their large size hinders the adsorption on the surface and the removal from the ready-made imprint [30]–[33].

The large number of binding sites and functional groups on the protein surface can increase problems related to non-specific binding with molecularly imprinted proteins (MIPs) leading to poor selectivity [34]. Therefore it is important to optimize all the required parameters and conditions to maintain the stability of such large structures throughout the process of imprinting. We selected BSA protein (65 kDa) for optimization process due to its similar size to cancer biomarker candidate HAPLN1. In Figure 3a and Figure 3b more stable imprints are formed with 12 hour incubation time and an increase in response occurs when more target molecules are imprinted at 30 µg/ml compared to 1 ng/ml concentration. Similar conditions were used by Wang et al [35] to show how imprints formed with different size proteins are highly specific to their respective imprint electrode using potentiometric detection. MALDI TOF technique has been used as a novel method in our work to confirm the specificity of the imprint site, also shown by potentiometric detection. Figure 4 shows myoglobin proteins bind specifically to its imprinted cavities and not to the BSA cavities. We believe that since myoglobin proteins are much smaller in size (17 kDa) compared to BSA, myoglobin would not fit in the BSA mold and hence not binding to larger BSA cavities. Results of BSA specificity could not be confirmed with MALDI-TOF due to the poor sensitivity of the equipment towards high molecular weight proteins. We have used potentiometric detection for optimization of cavities with BSA and for detection of HAPLN1 in buffer and in spiked serum. Our results in Figure 5a show 25 mV response of HAPLN1 to HAPLN1 imprinted electrode as compared to low response by BSA protein in buffer and similar results were obtained in spiked serum. Although we show the potentiometric response obtained is specific to the protein imprinted and is a result of protein binding, a clear understanding is currently lacking for the electrochemical signal (potential change) induced by protein adsorption. Several mechanisms have been suggested including by Thompson et al. He proposes factors, which might contribute to change in work function [36] of gold when protein binds to the gold surface and thus show how change in work function results in change of surface potential. In future experiments we plan to further check specificity of HAPLN1 protein binding to imprint cavities by surface plasma resonance technology (SPR) and also to obtain clinical data for validation.

## Conclusions

Our data demonstrate the use of SAM forming water-soluble hydroxylated alkanethiols to imprint biomacromolecules. BSA was shown to be an effective model protein to optimize the control parameters such as protein concentration, ratio of the molecular components, and the incubation time of the chip to downscale the process of imprinting for detection of low concentrations of proteins. Molecular Imprinting based biosensor was successfully built to recognize malignant pleural mesothelioma cancer biomarker HAPLN1 and demonstrated high sensitivity by detecting low concentration of biomarker in serum/buffer solution. The sensor showed a limit of detection of picomolar concentrations with a response time of 2–5 minutes. The low cost of the sensor is based on the lack of expensive monoclonal antibodies, additional labeling molecules and on the simplicity of the open circuit potential measurement with a potentiometer, which also is very easy to use (similar to pH meter).

## References

[1] Fux L, Ilan N, Sanderson RD, Vlodavsky I (2009) Heparanase: busy at the cell surface. Trends Biochem Sci 34(10): 511–519. 19733083 10.1016/j.tibs.2009.06.005 PMC2755511

[2] Zhang J, Yang J, Han X, Zhao Z, Du T, et al (2012) Overexpression of heparanase multiple antigenic peptide 2 is associated with poor prognosis on gastric cancer: Potential for therapy. Oncology Letters 4: 178–182. 22807984 10.3892/ol.2012.703 PMC3398369

[3] Liu H, Chen X, Gao W, Jiang G (2012) The expression of heparanase and microRNA-1258 in human non-small lung cancer. Tumor boil 33: 1327–1334. 10.1007/s13277-012-0380-9 22488243

[4] Okawa T, Naomoto Y, Nobuhisa T, Takaoka M, Motoki T, et al (2005) Heparanase is involved in angiogenesis in esophageal cancer through induction of cyclooxygenase-2. Clin Cancer Res 11(22): 7995–8005. 16299228 10.1158/1078-0432.CCR-05-1103

[5] Cohen-Kaplan V, Jrbashyan J, Yanir Y, Naroditsky I, Ben-Izhak O, et al (2012) Heparanase induces signal transducer and activator of transcription (STAT) protein phosphorylation. J. Biol. Chem. 287(9): 6668–6678. 10.1074/jbc.M111.271346 PMC3307274 22194600

[6] Cohen I, Pappo O, Elkin M, San T, Bar-Shavit R, et al (2006) Heparanase promotes growth, angiogenesis, and survival of primary breast tumors. Int J Cancer 118: 1609–1617. 16217746 10.1002/ijc.21552

[7] Quiros RM, Rao G, Plate J, Harris JE, Brunn GJ, et al (2006) Elevated serum heparanse-1 levels in patients with pancreatic carcinoma are associated with poor survival. Cancer 106(3): 532–540. 16388520 10.1002/cncr.21648

[8] Ramani VC, Yang Y, Ren Y, Nan L, Sanderson RD (2005) Heparanase plays a dual role in driving hepatocyte growth factor (HGF) signaling by enhancing HGF expression and activity. J Biol Chem 286(8): 6490–6499. 10.1074/jbc.M110.183277 PMC3057851 21131364

[9] Creaney J, Robinson BWS (2009) Serum and Pleural fluid biomarkers for mesothelioma. Current Opinion in Pulmonary Medicine 15: 366–370. 19417672 10.1097/MCP.0b013e32832b98eb

[10] Ropponen K, Tammi M, Parkkinen J, Eskelinen M, Tammi R, et al (1998) Tumor Cell-associated Hyaluronan as an Unfavorable Prognostic Factor in Colorectal Cancer. Cancer Res 58: 342–347. 9443415

[11] Anttila MA, Tammi RH, Tammi MI, Syrjänen KJ, Saarikoski SV, et al (2000) High Levels of Stromal Hyaluronan Predict Poor Disease Outcome in Epithelial Ovarian Cancer. Cancer Res 60: 150–155. 10646867

[12] Gotte M, Yip GW (2006) Heparanase, Hyaluronan and CD44 in Cancers: A Breast Carcinoma Perspective. Cancer Res 66: 10233–10237. 17079438 10.1158/0008-5472.CAN-06-1464

[13] Xu XM, Chen Y, Chen J, Yang S, Gao F, et al (2003) A Peptide with Three Hyaluronan Binding Motifs Inhibits Tumor Growth and Induces Apoptosis. Cancer Res 63: 5685–5690. 14522884

[14] Golshani R, Lopez L, Estrella V, Kramer M, Iida N, et al (2008) Hyaluronic Acid Synthase-1 Expression Regulates Bladder Cancer Growth, Invasion and Angiogenesis through CD44. Cancer Res 68: 483–491. 18199543 10.1158/0008-5472.CAN-07-2140

[15] Ivanova AV, Goparaju CMV, Ivanov SV, Nonaka D, Cruz C, et al (2009) Protumorigenic role of HAPLN1 and its IgV domain in malignant pleural mesothelioma. Clin Cancer Res 15: 2602–2611. 19351750 10.1158/1078-0432.CCR-08-2755 PMC3761224

[16] Lavignac N, Allender CJ, Brain KR (2004) Current status of molecularly imprinted polymers as alternatives to antibodies in sorbent assays. Analytica Chimica Acta 510: 139–145.

[17] Polyakov MV (1931) Adsorption properties and structure of silica gel. Zhur Fiz Khim 2: 799–805.

[18] Dickey FH (1949) Specific adsorbents. Proc Natl Acad Sci 35: 227– 229. 10.1073/pnas.35.5.227 PMC1063007 16578311

[19] Wulff G, Sarhan A (1972) Uber die Anwendung von enzyme analog gebauten Polymeren zur Racemattrennung, Angew. Chem 84: 364.

[20] Arshady R, Mosbach M (1982) Synthesis of substrate-selective polymers by host–guest polymerization, Macromol. Chem. Phys.-Makromol. Chem 182: 687–692.

[21] Porter MD, Bright TB, Allara DL, Chidsey CED (1987) Spontaneously organized molecular assemblies. J Amer Chem Soc 109: 3559–3568.

[22] Wang Y, Zhang Z, Jain V, Yi J, Mueller S, et al (2010) Potentiometric sensors based on surface molecular imprinting: Detection of cancer biomarkers and viruses. Sensors and Actuators B 146: 381–387.

[23] Janata J (1975) Immunoelectrode. J Amer Chem Soc 97: 2914–2916.

[24] Nelson GM, Padera TP, Garkavtsev I, Shioda T, Jain RK (2007) Source Differential gene expression of primary cultured lymphatic and blood vascular endothelial cells. Neoplasia 9(12): 1038–1045. 18084611 10.1593/neo.07643 PMC2137938

[25] Zhou YX, Yu B, Levon K (2005) Potentiometric sensor for dipicolinic acid. Biosensors & Bioelectronics 20: 1851–1855. 15681204 10.1016/j.bios.2004.05.005

[26] Aizenberg J, Black AJ, Whitesides GM (1999) Control of crystal nucleation by patterned self-assembled monolayers. Nature 398: 495–498.

[27] Mirsky VM, Hirsch T, Piletsky SA, Wolfbeis OS (1999) A spreader-bar approach to molecular architecture: formation of stable artificial chemoreceptors. Angewandte Chemie-International Edition 38: 1108–1110. 25138511 10.1002/(SICI)1521-3773(19990419)38:8<1108::AID-ANIE1108>3.0.CO;2-C

[28] Piletsky SA, Piletskaya EV, Sergeyeva TA, Panasyuk TL, Elskaya AV (1999) Molecularly imprinted self-assembled films with specificity to cholesterol. Sensors and Actuators B: Chemical 60: 216–220.

[29] Li X, Husson SM (2006) Two-dimensional molecular imprinting approach to produce optical biosensor recognition elements. Langmuir 22: 9658–9663. 17073493 10.1021/la0612163

[30] Du XZ, Hlady V, Britt D (2005) Langmuir monolayer approaches to protein recognition through molecular imprinting, Biosensors & Bioelectronics. 20: 2053–2060. 10.1016/j.bios.2004.08.044 15741075

[31] Das K, Penelle J, Rotello VM (2003) Selective picomolar detection of hexachlorobenzene in water using a quartz crystal microbalance coated with a molecularly imprinted polymer thin film. Langmuir 19: 3921–3925.

[32] Turner NW, Jeans CW, Brain KR, Allender CJ, Hlady V, et al (2006) From 3D to 2D: a review of the molecular imprinting of proteins. Biotechnology Progress 26: 1474–1489. 10.1021/bp060122g PMC2666979 17137293

[33] Bossi A, Bonini F, Turner APF, Piletsky SA (2007) Molecularly imprinted polymers for the recognition of proteins: the state of the art. Biosensors & Bioelectronics 22: 1131–1137. 16891110 10.1016/j.bios.2006.06.023

[34] Jeyachandran YL, Mielczarski JA, Mielczarski E, Rai B (2010) Efficiency of blocking of non-specific interaction of different proteins by BSA adsorbed on hydrophobic and hydrophilic surfaces. J Colloid Interface Sci 341: 136–142. 19818963 10.1016/j.jcis.2009.09.007

[35] Wang YT, Zhou YX, Sokolov J, Rigas B, Levon K, et al (2008) A potentiometric protein sensor built with surface molecular imprinting method. Biosensors & Bioelectronics 24: 162–166. 18514502 10.1016/j.bios.2008.04.010

[36] Huo H, Cheran LE, Thompson M (2007) Kelvin physics of protein layers printed in microarray format. ACS Symposium Series 312–337.

